# Appendiceal mucocele—A rare case report

**DOI:** 10.1016/j.ijscr.2019.04.008

**Published:** 2019-04-09

**Authors:** Sunil Kumar B.B., Pranav Jasuja

**Affiliations:** a Department of Surgical Gastroenterology, JSS Medical College and Hospital, Mysore, India; b Deprtment of General Surgery, JSS Medical College and Hospital, Mysore, India

**Keywords:** Mucocele, Appendix, Mucinous cystadenoma, Case report, Hemicolectomy

## Abstract

**Introduction:**

Appendiceal mucocele is a rare disease and has a clinical picture that resembles acute appendicitis. A correct diagnosis before surgery is very important for the selection of surgical technique(appendicectomy/colectomy)to avoid severe intraoperative and postoperative complications. The work has been reported in line with the SCARE criteria (Agha et al. [23]).

**Presentation of case:**

We present a case of 70 year old female admitted with chief complaints of right iliac fossa pain intermittent in nature with nausea and generalized weakness since 1 month. With the help of USG and CECT preoperative diagnosis of Appendiceal Mucocele was made. Midline incision Laparotomy was performed. Preoperative diagnosis of Appendix Mucocele changing the course of management has been reported rarely in literature.

**Discussion:**

Intraoperatively a cystic mass of appendix with dimensions 8 cm × 5 cm with broad base and inflamed walls communicating with caecum but without perforation was discovered in right iliac fossa. Extended right hemicolectomy with ileotransverse anastomosis was done. Histopathological diagnosis of Mucinous Cystadenoma with Mucocele was reported. After 6 months of surgery patient is doing well with no postoperative complications.

**Conclusion:**

In our opinion every patient more than 50 years old presenting in emergency department with clinical symptoms of acute appendicitis must undergo CT and open surgery should be favoured against laparoscopic surgery.

## Highlights

• A Mucocele appendix with wide base communicating with caecum is a rare presentation.
• Presentation is same as acute appendicitis.
• Pre-operative diagnosis even with the help of USG & CT is really a challenging issue.
• Diagnosis of wide base mucocele appendix (preoperatively/intraoperatively) changes whole course of surgical management.

## 1 Introduction

• The mucocele of the appendix was first described in 1842 by Rokitansky [1]. This disease is considered as a rare lesion of the appendix, which is found in 0.3 to 0.7% of the appendectomies [2]. It is characterized by the dilation of the organ lumen with mucus accumulation. Appendix mucocele may come as a consequence of obstructive or inflammatory processes, cystadenomas or cystadenocarcinomas [3]. Besides these causes, other tumor lesions in the appendix or cecum may present as mucocele [4]. Its Main complication is pseudomyxoma peritonei.The work has been reported in line with the SCARE criteria (Agha et al. [23]).

## 2 Case report

**Fig. 1:**
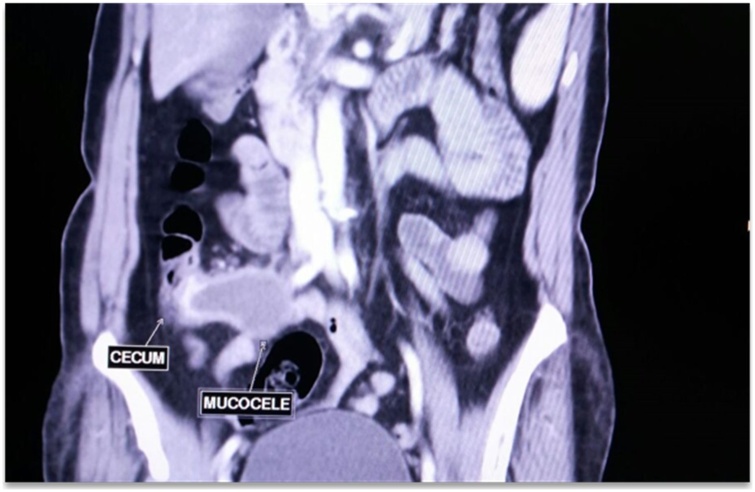
CECT image of mucocele appendix, with wide base and communicating with caecum.

**Fig. 2:**
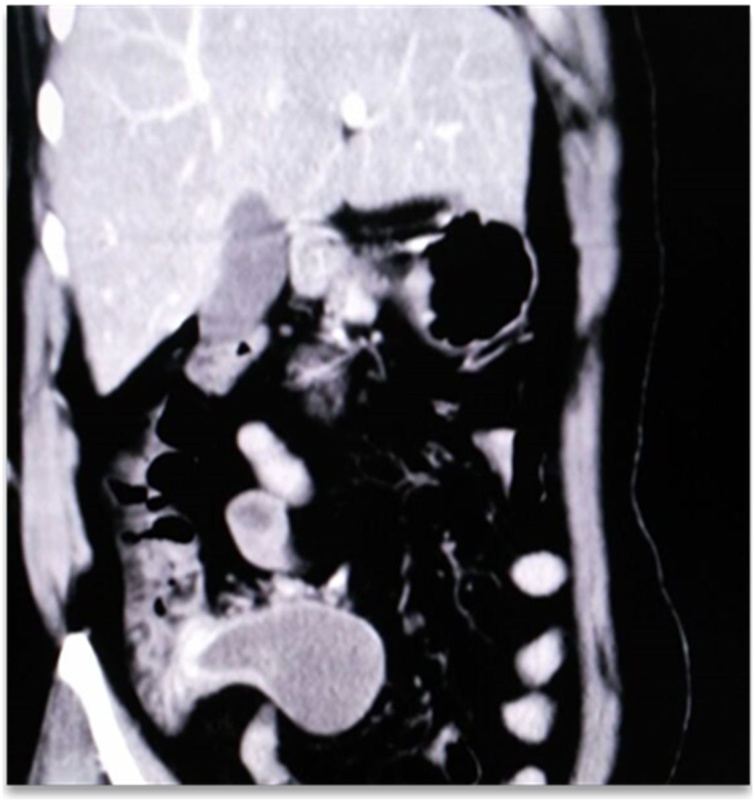
CECT image of mucocele appendix, with wide base and communicating with caecum.

**Fig. 3:**
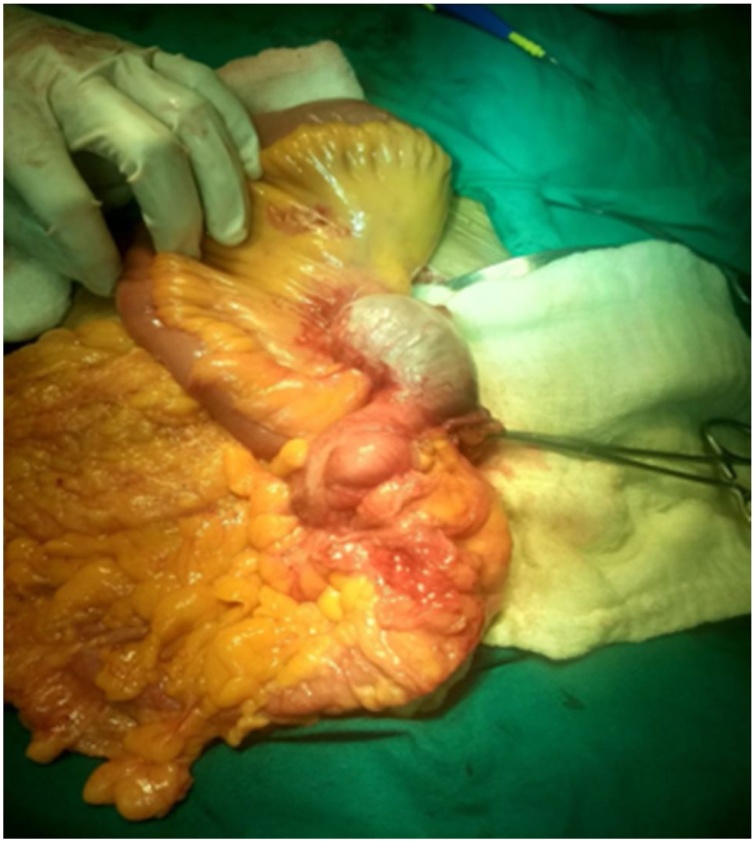
Intraoperative pictures of mucocele appendix.

**Fig. 4:**
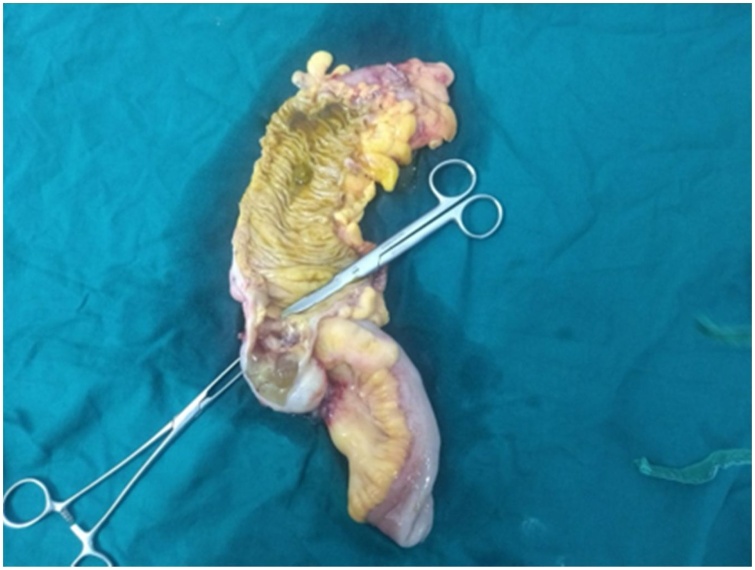
Hemicolectomy specimen showing wide base appendix communicating with caecum.

**Fig. 5:**
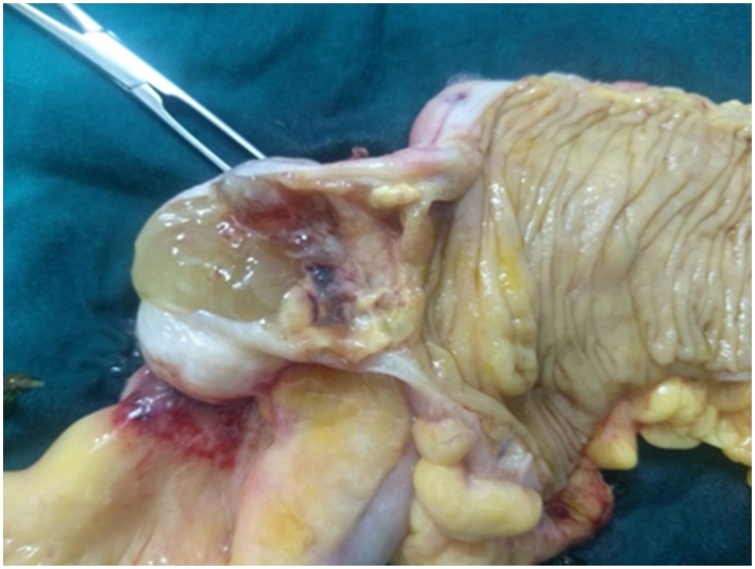
Macroscopic view of mucocele appendix with mucous as content.

A 70 year old female came to Out Patient Department of Department of Surgical Gastroenterology, with 1 months history of vague pain abdomen, more localized to right lower abdomen, associated with generalized weakness, nausea and decreased appetite from last 6 months, no history of surgeries in the past. Patient reported mild right iliac fossa tenderness on palpation. She was afebrile. Laboratory investigations showed Leucocytosis with neutrophilia. Abdominal Ultrasonography showed encapsulated cystic lesion in the lower quadrant of the abdomen with a liquid content of variable echogenicity -? Appendicular Abscess /Mucocele Appendix. Abdominal CECT was done which showed Well circumscribed low attenuating tubular mass contiguous with the base of the caecum showing thin curvy linear mural calcifications with few low attenuating areas along the surface of the lesion f/s/o Mucocele of Appendix (Fig. 1, Fig. 2). Vertical Midline Incision Laparotomy was performed. Intraoperatively a cystic mass of appendix with dimensions 8 cm × 5 cm with broad base and inflamed walls communicating with caecum but without perforation was discovered in right iliac fossa. Multiple significant lymph nodes of mesoappendix and ileocolic region were also found. With suspicion of malignancy and non-availability of frozen section, Extended right hemicolectomy with ileotransverse anastomosis was done (Fig. 3, Fig. 4, Fig. 5). Histopathological diagnosis of Mucinous Cystadenoma with Mucocele was reported. After 6 months of surgery patient is doing well with no postoperative complications.

## 3 Discussion

The mucocele of the appendix - The cystic dilation of the appendix caused by the accumulation of mucus secretion. This process is slow and gradual, with no signs of infection inside the organ. It results from the lumen obstruction in the appendix, which is secondary to the inflammatory or neoplastic proliferation of the appendix mucosa, or of lesions in the cecum, adjacent to the appendiceal ostium. While some articles confirm its prevalence among women [5,6], others demonstrate a higher incidence among men [7,8].

**Fig. 6:**
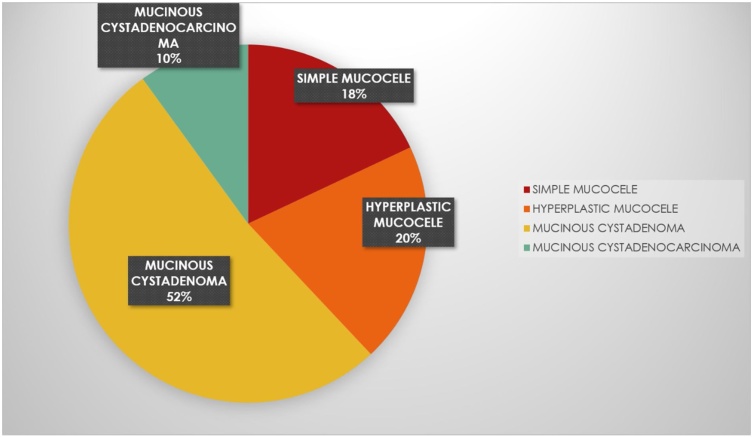
Pie chart showing histological classification of mucocele appendix with percentage prevalence.

Mucocele in the appendix may be classified according to the histological characteristics of lumen obstruction [9] (Fig. 6)

• **Simple mucocele** (inflammatory, obstructive or retention cyst) -degenerative epithelial changes and results in the obstruction and the distension of the appendix. There is no evidence of hyperplasia or mucosal atypia.
• **In hyperplastic mucocele**, the appendix dilation occurs due to the hyperplastic growth of the appendix or cecal mucosa, just like hyperplastic polyps in the colon.
• **The mucinous cystadenoma** is an appendix neoplasm with dysplastic epithelium similar to colon adenomatous polyps
• **The mucinous cystadenocarcinoma** presents high grade cellular dysplasia and stromal invasion, besides muscularis mucosae
• In both types described, the mucus material contains epithelial adenoma cells with low or high grade of dysplasia. The rupture of the appendix may lead to the dissemination of the epithelium that produces mucins in the abdominal cavity, causing mucinous ascites or **pseudomyxoma peritonei**.

The clinical flow of the disease does not have a specific picture. It often flows asymptomatically. In about 50% of cases it is discovered accidentally during radiologic and endoscopic examinations or at surgery. A patient’s clinical symptoms may include pain in the right lower quadrant of the abdomen, palpable abdominal mass, nausea, vomiting, weight loss, gastrointestinal bleeding, and signs of intussusception of the intestines. Preoperative diagnosis of appendicular mucocele is very important for the selection of an adequate surgical method to prevent peritoneal dissemination, to prevent intraoperative and postoperative complication, and repeated surgery [10,11]. USG, computed tomography (CT), and colonoscopy is used for diagnostics. USG is the first-line diagnostic method for patients with acute abdominal pain. USG can be used to differentiate between mucocele and acute appendicitis. In case of acute appendicitis, the outer diameter threshold of the appendix is 6 mm, and 15 mm and more indicates the presence of a mucocele, with 83% sensitivity and 92% specificity [[12], [13], [14], [15]]. CT is regarded as the most accurate method of diagnostics. CT can be used to discover the signs specific to mucocele with high accuracy: appendix lumen more than 1.3 cm, its cystic dilatation, and wall calcification. By colonoscopy an elevation of the appendiceal orifice is seen and a yellowish mucous discharge would be visible from this orifice. One of the cardinal principles of surgical treatment of this disease is that intact mucoceles do not pose a threat for the patient. If it is perforated and the filling turns up in the peritoneal cavity, there is a high probability that pseudomyxoma peritonei will develop, for which treatment is very problematic and long-term results are quite unsatisfactory. Therefore, the selection of an adequate surgical method is very important. Some surgeons think that open surgery should be favored against laparoscopy. If the surgery was launched using a laparoscopic method and it appears that there is an appendiceal mucocele, it must be converted into open surgery. This has 2 objectives: (1) to perform surgery carefully so the cyst is not ruptured and the filling is not scattered into the peritoneal cavity and (2) with an open surgery compared to the laparoscopic method, it is possible to have a fuller inspection, palpation, and direct inspection of the spots in the abdomen where mucinous tumors are most common.

An algorithm for the selection of the type of surgery has been furnished by Dhage-Ivatury and Sugarbaker [16]. It envisages several factors:

(1) whether or not a mucocele is perforated;
(2) whether the base of the appendix (margins of resection) is involved in the process; and
(3) whether there are positive lymph nodes of mesoappendix and ileocolic

**Fig. 7:**
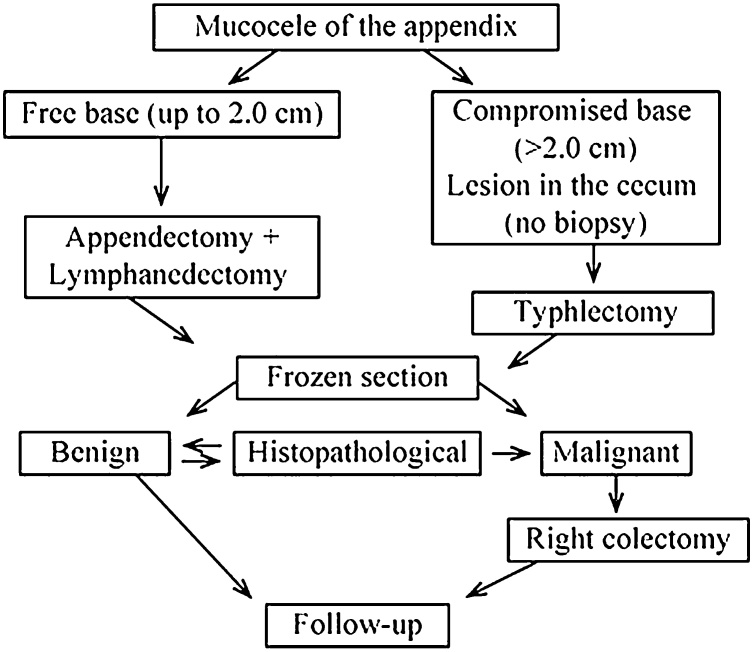
Treatment protocol for mucocele appendix.

As a result patients may require different operations: appendectomy to the right colectomy, including cytoreductive surgery, heated intraoperative intraperitoneal chemotherapy, early postoperative intraperitoneal chemotherapy [16]. In our case mucocele appendix was not perforated with involvement of base and positive lymph nodes, so extended right hemicolectomy was performed. Another protocol has been suggested recently based on intraoperative findings(base), frozen section and histopathology (Fig. 7).

Treatment of pseudomyxoma peritonei is variable, both due to the rarity of the disease and to its frequently slow-growing nature [17]. Current treatment strategies range from watchful waiting to extensive cytoreductive surgery alone or with hyper thermic intraoperative peritoneal chemotherapy (HIPEC) or early postoperative intraperitoneal chemotherapy (EPIC) [18]. Based on the Sugarbaker peritonectomy procedure, a recent study showed that cytoreductive surgery with intraperitoneal hyper thermic perfusion permitted complete tumor removal, confirming the efficacy of this combined treatment in terms of improved long-term survival and better regional control of the disease [19]. However, other studies support that fluorouracil-based adjuvant systemic chemotherapy should be the standard of care for patients with PMP of appendiceal origin [20]. In situations where surgery is not immediately required, patients can be monitored via CT scans, tumor marker laboratory tests, and physical symptoms, to determine when, and if, surgery is warranted. Since the risk of developing an adenocarcinoma of the colon is 6 times greater in patients with a mucocele than in the general population, colonic surveillance is warranted in these cases [21]. The prognosis of patients with pseudomyxoma peritonei was very poor, with limited life expectancy and no chances of healing. The cytoreduction associated with hyper thermic intraperitoneal chemotherapy has reached survival rates in five years of 50% to 96%, in selected cases, when peritoneal cytoreduction is complete and there are no distant metastases [22].

## 4 Conclusion

Appendiceal mucocele is a rare disease and has a clinical picture that resembles acute appendicitis. A correct diagnosis before surgery is very important for the selection of surgical technique to avoid severe intraoperative and postoperative complications. USG, particularly CT, should be used extensively for this purpose. In our opinion, every patient more than 50 years old who arrives at the emergency department with clinical symptoms of acute appendicitis must undergo CT and open surgery should be favored against laparoscopic surgery.

## Conflicts of interest

The authors have no conflict of interest to disclose.

## Sources of funding

No source to be stated.

## Ethical approval

For case report our Institute exempted to take ethical approval.

## Consent

Written informed consent was obtained from the patient for publication of this case report and accompanying images. A copy of the written consent is available for review by the Editor-in-Chief of this journal on request.

## Author contribution

Pranav Jasuja – Wrote the manuscript.

Sunil Kumae B.B. – Operated the patient.

## Registration of research studies

This is not a study, but a case report.

## Guarantor

Dr. Pranav Jasuja.

## Provenance and peer review

Not commissioned, externally peer-reviewed.
